# Clinical Lecture on a Case of Insular Sclerosis in a Child

**Published:** 1901-03-23

**Authors:** Dyce Duckworth

**Affiliations:** Physician and Lecturer on Medicine at St. Bartholomew's Hospital


### Hospital Clinics and Medical Progress.

### CLINICAL LECTURE ON A CASE OF INSULAR SCLEROSIS IN A CHILD.

Under the care of Sir Dyce Duckworth, M.D., F.R.C.P., Physician and Lecturer on Medicine at St. Bartholomew's Hospital.

(*Specially reported for " The Hospital."*)

This little boy was admitted only five or six days ago. He is seven years old, and came here on account of certain trembling movements which were noticed. The symptoms are not very well marked, but the shakings increase very much when he attempts to do anything. When he rises up from the bed his head oscillates to and fro. You see he is pale and rather small for his age. The knee-jerks are very brisk; there is nothing very remarkable about the gait though there is some spasticity in the legs. He was late in beginning to walk, and he has had this shaking of the head, hands, and feet since he was one month old. He has always walked with his toes turned inwards. He has three brothers who are quite healthy. I will get him to write something so that you may see a specimen of his handwriting, which is very characteristic of the disease. You would, not knowing the circumstances under which it was written, imagine it to have been done by a man in delirium tremens. You will also notice that he has to bring the other hand on to his right hand to keep it as still as possible, but with only partial success. While standing still he is practically steady, but directly he wants to *do* anything the shaking begins.

The cardinal symptoms of this disease are better seen in the adult than in the child, and it is certainly more common in the former than in the latter. My own personal experience of this disease in children is very limited. It occurs between the ages of 20 and 40, and to particularise still farther, it generally is most often present in the thirties. Both sexes are attacked, but it is slightly more common in men than in women; but children are said to be not infrequently the victims of it. The symptoms are the presence of intention or volitional tremors; the scanning of the speech, which is very peculiar if the disease is well marked—a slow, staccato sort of speech. The gait is stiff or "spastic," the knee-jerks are increased and nystagmus is present. This little patient has nystagmus, though it is not particularly well marked. His case, I must tell you, is not complete, all the classical symptoms not being well marked, but it possesses all the essential factors, which enable one to be sure of one's diagnosis. You must not always expect to find "samples" of diseases, corresponding exactly in every detail with the descriptions of the text-books. Constantly one or more symptoms are dropped out of the category; then you must make your diagnosis from the presence of symptoms that are sufficient to show you the type of the disorder you are dealing with. Just as in Graves's disease, you know the three car linal symptoms are goître, prominent eyeballs, and great rapidity of pulse. If you had in a case, any two of these three symptoms, you would call it Graves's disease, and, indeed, one may often diagnose Graves's disease from the presence of tachycardia only. The important thing to do is to look out for the type of case, and I cannot impress this upon you too strongly. To take up our subject again, insular sclerosis is known to be dependent on little "blights" that have exerted their influence on the cerebro-spinal system. These blights occur in little areas of nerve-matter which have been broken up and replaced by connective tissue—little islands or islets; hence the term *insular* in the nomenclature. So we have these "islets of hard connective tissue" forming little breaks in the lines of the nervous connection, and they cause stoppages in the passage of impressions up and down, which are outwardly manifested by the trembling nature of the movements. Their origin is unknown. The disease is supposed to follow upon acute infectious diseases, such as scarlet fever and measles. If this is the case it must be a very uncommon result of these disorders! Practically we very rarely see it as a sequel. Influenza has been described as the cause in some cases, but nowadays so much is ascribed to influenza that we have to receive with reserve this explanation of the ætiology. In the light of to-day the bacteriological factor has to be considered; a suspicion exists that the causation may be due to certain toxins which exert their influence in these islets and patches, and the same idea is now somewhat prevalent with respect to anterior poliomyelitis (acute), which is considered to be in many respects analogous to insular sclerosis. Be that as it may, the common result is that cases of insular sclerosis go from bad to worse, and that the disease is uninfluenced by drugs. The only thing to do is to keep the patients in good health, to feed them well, and keep them warm. The disease has remissions, the patients improving and then relapsing. Quinine, belladonna, arsenic, and silver nitrate are the drugs most in vogue, and are supposed to do good: this is all that we can say of the influence of drugs upon the disease—*i.e.*, that they are supposed to do good. Such a malady occurring in any member of the family is truly distressing. It is always a sad thing to see young lives spoilt by ailments of this kind; but the marvel is that, out of the millions of human beings that come into this world, by far the greater majority arrive with their organs perfect and ready to perform their functions so well as they do. While these blights and partial abortions of nature are happily the exception, though there are plenty of them, the day may come when their ætiology will be understood. With increased knowledge in every direction comes increased knowledge of treatment and the prevention and removal of maladies. We shall keep the patient in and give him quinine, belladonna, and a course of silver nitrate, taking care not to give him argyria, *i.e.* not to stain him for the rest of his days. We are justified in using all means to promote the general health in these cases, because, so far as I have observed, we have met with the best results from this course.

